# A demonstration of an affinity between pyrite and organic matter in a hydrothermal setting

**DOI:** 10.1186/1467-4866-12-3

**Published:** 2011-02-07

**Authors:** Paula Lindgren, John Parnell, Nils G Holm, Curt Broman

**Affiliations:** 1 Department of Geographical and Earth Sciences, University of Glasgow, Glasgow G12 8QQ, UK; 2 Department of Geology and Petroleum Geology, University of Aberdeen, Aberdeen AB24 3UE, UK; 3 Department of Geological Sciences, Stockholm University, 106 91 Stockholm, Sweden

## Abstract

One of the key-principles of the iron-sulphur world theory is to bring organic molecules close enough to interact with each other, using the surface of pyrite as a substrate in a hydrothermal setting. The present paper explores the relationship of pyrite and organic matter in a hydrothermal setting from the geological record; in hydrothermal calcite veins from Carboniferous limestones in central Ireland. Here, the organic matter is accumulated as coatings around, and through, pyrite grains. Most of the pyrite grains are euhedral-subhedral crystals, ranging in size from ca 0.1-0.5 mm in diameter, and they are scattered throughout the matrix of the vein calcite. The organic matter was deposited from a hydrothermal fluid at a temperature of at least 200°C, and gives a Raman signature of disordered carbon. This study points to an example from a hydrothermal setting in the geological record, demonstrating that pyrite can have a high potential for the concentration and accumulation of organic materials.

## Introduction

An important requirement for the origin of life is the concentration of organic compounds to allow interaction with each other and with other chemical species. An efficient mechanism for concentrating organic molecules is the adsorption onto mineral surfaces. There are several examples of minerals with strong affinities for the accumulation of organic matter. These include for instance clays [1], radioactive minerals [2], zeolites and feldspars [3] and sulphide minerals including pyrite (FeS_2_) [4,5].

Here, we study the relationship between pyrite and migrated organic matter in the geological record, via hydrothermal deposits of the Irish Carboniferous, to assess the potential of pyrite acting as a substrate for organic matter.

### Pyrite and the iron-sulphur world

The iron-sulphur world hypothesis for the origin of life was proposed by Wächtershäuser [5]. He suggests that the formation of pyrite (FeS_2_) is the first energy source for life. The iron-sulphur world takes place in a hydrothermal setting, where iron- and sulphur-rich water produces abundant pyrite deposits. The formation of pyrite releases energy that could have been utilized during an autotrophic setting for the origin of life. The iron-sulphur world scenario for the origin of life stands in contrast to the heterotrophic origin of life in the "cold soup" theory [6,7]. In addition to an autotrophic origin of life, the iron-sulphur world also proposes pathways for the origin of cell membranes, the origin of DNA, and a range of other biochemical reactions essential to the origin of life [8].

A key-aspect of the iron-sulphur world theory is the adsorption of organic molecules to the surface of pyrite. The theory predicts that since the surface of pyrite is slightly positively charged, it would attract and bind negatively-charged organic molecules. The organic molecules would be connected to the surface of pyrite with weak ionic bonds, and therefore able to migrate rather freely around the surface of the pyrite crystal. This would make them more likely to interact and form more complex molecules. In fact, pyrite has a positive net charge in acid conditions below pH 6.8, which would be similar to conditions in an oxygen-depleted primordial environment, and a negative charge above pH 6.8 [9]. Several of the chemical reactions involved in the iron-sulphur world scenario have been tested successfully in the laboratory [9-13]. For example, pyrite strongly adsorbs adenine (one of the most important organic molecules for life) in a medium that simulates primordial aqueous environments, and the adsorption of adenine is enhanced in the presence of acetate (an organic precursor of complex metabolic pathways) and in an oxygen-depleted environment [9,10].

To explore the relationship of pyrite and organic matter in a hydrothermal setting from the geological record, we have studied Carboniferous hydrothermal calcite veins from Mullaghwornia, Ireland, at a locality which retain both pyrite and organic matter.

### Pyrite and organic matter in the Irish Carboniferous

**Figure 1:**
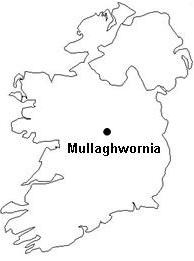
**Outline map of Ireland showing the location of Mullaghwornia quarry at Ballymahon, central Ireland**.

The Carboniferous rocks of Ireland contain numerous sulphide ore deposits, which also at some localities include visible accumulations of organic matter [14], and in some cases concentrations of up to 8.6% organic carbon [15]. The sulphide deposits formed during late Devonian to early Carboniferous, about 340 Ma ago, when Ireland suffered from rifting and extension during the break-up of an old red sandstone continent. This event was accompanied by widespread volcanism and the production of hydrothermal ore deposits, including the sulphides [16]. Also, at this period, a marine transgression gave rise to extensive carbonate deposition. These sediments, including bituminous limestone and black shale, are the most likely source of the organic matter in the sulphide deposits [14]. The sulphide deposits were precipitated widely in the Irish Carboniferous limestone terrain, and the sulphur was derived either from bacterial sulphate reduction of seawater, or from an abiogenic deep-seated hydrothermal source [17]. However, a study of sulphur isotopes from the orebody at Navan, central Ireland, shows that bacteria were responsible for the majority of the sulphide deposition there [18]. The sample locality for this study is the Mullaghwornia quarry at Ballymahon, central Ireland (Figure 1). This is an abandoned quarry exposing Dinantian (Lower Carboniferous) limestone, with a zone of hydrothermal calcite veining, containing pyrite and organic matter, cross-cutting a limestone-host.

## Results and discussion

### Composition of hydrothermal veins from Mullaghwornia

**Figure 2:**
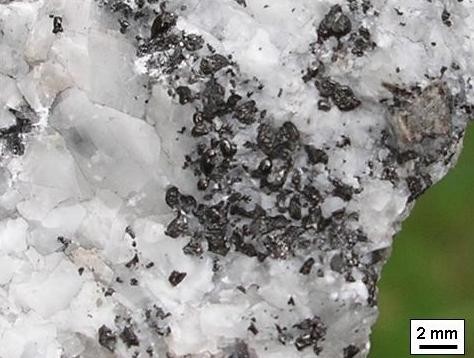
**Hydrothermal calcite vein with globules of organic matter (black) from Mullaghwornia quarry**.

The fine-grained grey limestone at the Mullaghwornia quarry (Figure 1) is cross-cut by a zone of branching hydrothermal mineral veins. These mineral veins belong to a single large complex calcite vein, but the individual veins are typically a few cm in thickness (Figure 2). The veins are primarily composed of ca 5 mm in diameter sized euhedral calcite (CaCO_3_) crystals, but they also contain pyrite (FeS_2_) and abundant solid migrated organic matter (bitumen).

**Figure 3:**
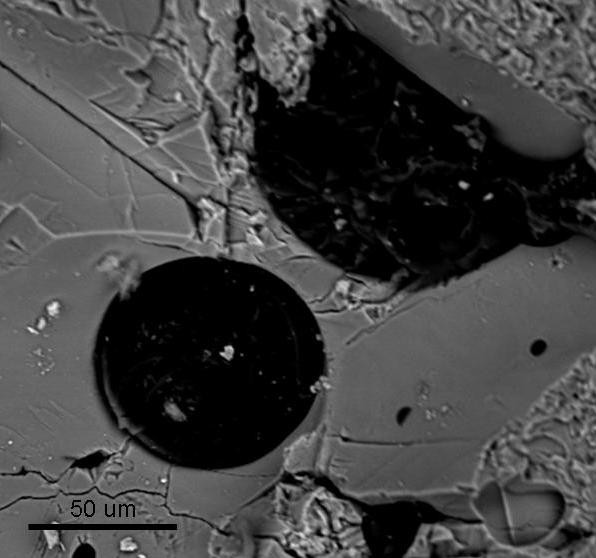
**Backscattered SEM micrograph of a vein sample showing organic matter (black) appearing as immiscible globules with curved boundaries within the calcite (grey)**.

The pyrite crystals range in size from ca 0.1-0.5 mm in diameter and are scattered throughout the matrix of the calcite veins. The pyrite occurs as euhedral-subhedral cubes, or parts of cubes. In some instances, the pyrite is brecciated with another sulphide phase, e.g. sphalerite (ZnS). The organic matter appears as coatings around the pyrite crystals, but also as immiscible globules with curved boundaries in the calcite (Figure 3). The immiscibility morphology between the calcite and the organic matter suggests that the fluid precipitating the calcite, and the fluid precipitating the organics, co-existed. An alternative explanation is that the organic matter here may be secondary infillings, filling in dissolution cavities in the calcite, in which case the fluid that deposited the organic matter is later than the fluid that deposited the calcite. Further, in some instances the organic matter is cross-cutting the calcite crystals, here also suggesting a later deposition of the organics. The relationships are not straightforward, but most likely it was several pulses of hydrothermal fluids that went through the vein; initially fluids precipitating calcite and pyrite, followed by later fluids precipitating organic matter filling in dissolution cavities in the pre-existing calcite and forming coatings around the pyrite crystals, or later co-existing immiscible fluids precipitating more calcite and organics simultaneously. The source of organic matter is unknown, but probably it is derived from the surrounding sedimentary terrain, where the hot hydrothermal fluids generated the migration of organics.

### Interaction of pyrite and organic matter

The occurrence of organic matter in hydrothermal ore deposits, including in sulphide deposits, is widespread [19]. Pyrite is the Earth's most abundant sulphide and occurs in a range of geological environments such as hydrothermal, sedimentary, and igneous settings. Pyrite regularly occurs as inclusions in migrated solid organic matter (bitumen). This relationship of pyrite and organics is not always straightforward, but can arise during direct precipitation and growth of the pyrite out of the bitumen [20]. It could also be an effect of the abrasion of pyrite from the surrounding rock by the migrating organics [21]. Pyrite in bitumen, and in other settings, can form both abiogenically or biogenically. Biogenic pyrite can form by sulphate-reducing bacteria producing H_2_S from sea-water sulphate, where the S^2- ^may be incorporated into pyrite, or it could form from the reduction of sulphur released by biological degradation of the migrated bitumen itself. Pyrite framboids, raspberry-like aggregates of pyrite spheres, are also sometimes found in organic matter. It is believed that most pyrite framboids are abiogenic and indicators of fast crystal growth, but pyrite framboids can also be a result of microbial activity [22].

**Figure 4:**
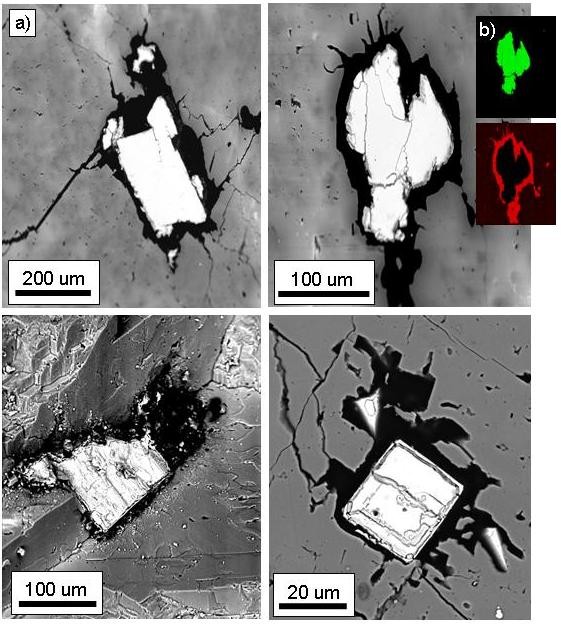
**Pyrite crystals coated with organic matter**. A) Backscattered SEM micrographs of pyrite crystals coated with organic matter in hydrothermal calcite veins from Mullaghwornia. The pyrite crystals appear white, the organic matter is black, and the calcite is grey. B) ED X-ray maps for sulphur (green) and carbon (red), with corresponding micrograph of pyrite and carbon.

**Figure 5:**
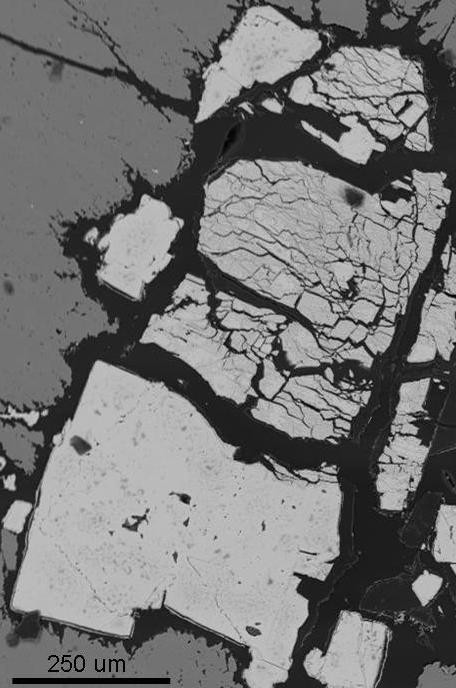
**Backscattered SEM micrograph of a pyrite crystal coated with organic matter**. In some cases the organic matter is invasive, i.e. penetrating and fracturing the pyrite crystals.

Here, in the hydrothermal setting of Mullaghwornia, there is indeed evidence of pyrite acting as an attractive substrate for the collection of organic matter; the majority of the pyrite crystals in the samples have organic coatings., Our observations are that twenty-four out of twenty-six observed pyrite crystals occurring in six blocks and thin sections from two different branches of the vein are coated with organic matter. The organic coatings are generally about 5-50 μm thick (Figure 4). In some cases the organic matter is invasive, i.e. penetrating and fracturing the pyrite crystals (Figure 5). This relationship makes it clear that the pyrite pre-dates the organic matter, i.e. the organics entered as a later phase than the pyrite and were subsequently adsorbed onto the surface of a pre-existing pyrite crystal. The sulphur isotopic composition, δ^34^S (^32^S/^34^S), of pyrite from Mullaghwornia is measured as -1.9 ‰ [20]. The composition of early Carboniferous seawater is about +15 ‰ [23]. The difference between the δ^34^S of early Carboniferous seawater and the δ^34^S of the Mullaghwornia pyrite gives a ΔS value of +17 ‰. Unless the sulphate pool was restricted and closed, this value is indicative of a non-biological fractionation process, i.e. the pyrite here probably has a deep-seated hydrothermal origin [24].

### Temperature of hydrothermal fluid

The temperature condition of the hydrothermal fluid which precipitated the Mullaghwornia calcite was deduced through fluid inclusion microthermometry [25]. The calcite contains numerous primary, randomly distributed, aqueous fluid inclusions. The inclusions have a poorly developed negative crystal (rhombohedra) shape and their size varies from 3 to 22 μm. The inclusions are two-phased with a vapor and a liquid phase. There were no oil-bearing fluid inclusions in the studied calcite. Microthermometric analysis was made on a total of nine aqueous fluid inclusions and the results are presented in Table 1. The homogenization temperature (Th) was measured in the range 200°C to 275°C, with homogenization to liquid in all inclusions. The Th-range provides minimum temperatures for the deposition of the Mullaghwornia calcite, as no correction for an unknown pressure could be taken into account. The homogenization temperatures from the Mullaghwornia calcite are within the higher portion of temperatures obtained from a number of other Irish carbonate-hosted ore deposits, where the temperatures range between 170°C to 240°C [26]. After freezing the Mullaghwornia inclusions, the first observed melting of ice (Tfm) occurred around -48°C to -50°C. This coincides with the eutectic temperature for the CaCl_2_-H_2_O system [25]. The final ice melting temperature (Tm) took place between -15.0°C and -16.2°C, and corresponds to a salinity of about 18.6 to 19.5 wt. % NaCl eq. [27]. The Irish carbonate-hosted ore deposits in [26] have a salinity of 12 to18 wt% NaCl. Thus, the Mullaghwornia calcite was deposited from a relatively highly saline hydrothermal CaCl_2_-dominated fluid at a temperature of at least 200°C, up to 275°C. The Mullaghwornia locality is located in a limestone terrain, which gives ground waters that are oversaturated in calcium, and hence a CaCl_2 _brine [28].

**Table 1:** Results of fluid inclusion microthermometry on fluid inclusions in calcite from a hydrothermal vein at Mullaghwornia.

Sample locality	Inclusion size (μm)	Ts (°C)	Tfm (°C)	Tm (°C)	Th (°C) (to liquid)
Mullaghwornia calcite	12	-63	-49	-15.2	264
	12	-66	-50	-15.6	264
	14	nd	-48	-15.0	276
	22	-63	-48	-15.0	200
	15	-63	-50	-15.3	246
	12	-63	-48	-15.6	229
	7	nd	nd	nd	205
	6	-72	-49.8	-16.2	214
	3	nd	nd	nd	223

Temperature abbreviations used: Ts = solidification, Tfm = first observed melting, Tm = final ice melting, Th = homogenization.

### Crystallinity of carbon

**Figure 6:**
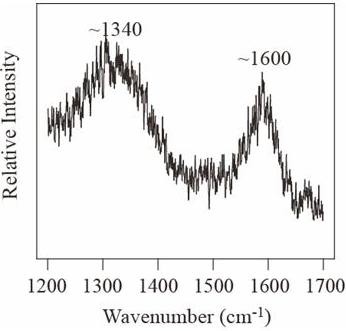
**The Raman signature of carbon in the hydrothermal vein is poorly crystalline, with a merged G+D2 peak at around 1600 cm**^**-1**^**, and a D1 peak at around 1340 cm**^**-1**^.

The fluid inclusion data reveals that the temperature of the hydrothermal fluid, and hence the temperature that the organic matter was exposed to, was at least 200°C, and up to 275°C. The higher temperature the organics are subjected to, the higher the degree of order of the carbon (more graphitic), with exception of carbon included in a melt [29]. Temperatures of 200°C to 275°C should not be enough for graphite to form [30], thus we expect the carbon in the hydrothermal vein to be disordered. Raman spectroscopic analysis is used on carbonaceous matter to determine if the carbon is disordered or graphitic [31]. The Raman spectrum (in the first order region) of carbonaceous matter includes a graphite peak (G peak) at around 1580 cm^-1^. Disordered carbon also contains a peak at around 1360 cm^-1 ^(D1 peak) and a peak at around 1620 cm^-1 ^(D2). The D2 peak appears as a right shoulder on the G peak, and in more disordered carbon it merges together with the G peak, causing a broad G + D2 peak around 1600 cm^-1^. The organic matter in the hydrothermal vein from Mullaghwornia gives a signature of poorly crystalline carbon (Figure 6); it has a D1 peak at around 1300 cm^-1^, and a merged G and D2 peak at around 1600 cm^-1^.

### Implications for sulphide substrates on Mars and the early Earth

This study is an example from the geological record showing that pyrite, the most abundant terrestrial sulphide, can act as a substrate for the concentration of organic matter, and that this evidence can be preserved and detected in the geological record. Sulphide substrates could also be important for carbon fixation on Mars. There is widespread evidence that sulphur species are prominent in Martian surface environments, assumed to have been introduced to the surface through volcanic activity [32]. The formation of the Mullaghwornia pyrite and organic matter is not a surface process, but it shows that if organic matter is available, it has an affinity to be preserved around pyrite. Currently, the Martian surface is highly oxidizing and therefore sulphates predominate, but early in the planet's history reducing conditions pertained. Accordingly, it has been suggested that sulphides occur on Mars [33], now preserved at depth. Sulphides are also known to be present on Mars from Martian meteorites [34,35]. Sulphide grains which are rimmed by a thin layer of poorly graphitized carbon have already been detected in carbonaceous chondrites and chondritic interplanetary dust particles [36,37], and in the Tagish Lake meteorite [38]. Other evidence from the geological record that sulphides preserve organics includes sulphides in Proterozoic and Ordovician sandstones from Canada, which contain high concentrations of amino acids [39].

In addition to acting as a substrate for the concentration of organic matter, there are also other advantages of sulphides as a potential target for the detection of life. Since sulphides that are produced by microbial sulphate reduction can preserve both morphological and chemical evidence of fossil life, they are proposed as targets for the exploration for fossil life on Mars, through measurements of isotopic composition [40] and search for entrapped microbial fossils [41].

The mineralogical composition of the early Earth is debated, but metal-sulphides were probably widespread [42], and in particular iron-sulphides are found as detrital mineral components in several Archaean deposits. In terms of the iron-sulphur world theory, the relationship of the pyrite and organic matter in the hydrothermal veins of Mullaghwornia is an example from the geological record supporting the idea that the surface of pyrite acts as a substrate for the adsorption of organic molecules.

### Experimental methods

The samples were prepared as thin sections and blocks for petrographic microscopy and for analyses with an XL30 environmental scanning electron microscope with a field emission gun (XL30 ESEM-FEG). The ESEM was equipped with an Oxford x-act energy dispersive spectrometer (EDS), backscatter electron detector (BSE) and secondary electron detector (SE). The samples were coated with a layer of ca 15 nm carbon prior to analyses in high vacuum. The acceleration voltage was 20 kV. The instrument was calibrated with a cobalt standard.

A 150 μm thick doubly-polished wafer was prepared for fluid inclusion analysis. The fluid inclusion microthermometric analyses were performed with a Linkam THM 600 stage, mounted on a Nikon microscope, with a working range from -196°C to +600°C. The thermocouple readings were calibrated by means of SynFlinc synthetic fluid inclusions and well-defined natural inclusions in Alpine quartz.

Raman spectroscopic analyses were conducted on thin sections and on small unpolished samples. Raman spectra were acquired by using a multichannel Dilor XY Laser Raman spectrometer. The laser source was an Innova 70 argon laser with a wavelength of 514.5 nm (green line). Laser focusing on the sample was performed through a petrographic microscope fitted with a 100× objective. The laser power was set at 200 mW at the entrance of the microscope. The spectra were accumulated in 20 increments with a measuring time of 3 s each. Calibration was made with respect to wavenumber using a neon laser and a silicon standard.

All the analyses were conducted at the Department of Geological Sciences, Stockholm University, Sweden.

## Competing interests

The authors declare that they have no competing interests.

## Authors' contributions

PL carried out the sampling, the majority of the analytical work and drafted the manuscript. JP, NGH and CB helped with analytical work and to draft the manuscript. All authors read and approved the final manuscript.
